# Cholangioscopy-assisted endoscopic mucosal resection for bile duct lesions through papillary support: A pilot exploration for super minimally invasive surgery in a porcine model

**DOI:** 10.1055/a-2208-5518

**Published:** 2023-12-11

**Authors:** Wengang Zhang, Ningli Chai, Bo Zhang, Zhenyu Liu, Jiafeng Wang, Qingzhen Wu, Enqiang Linghu

**Affiliations:** 1 Gastroenterology, The First Medical Center of Chinese PLA General Hospital, Beijing, China; 2 Department of Gastroenterology, The First Medical Center of Chinese PLA General Hospital, Beijing, China; 3 Chinese PLA General Hospital, Beijing, China; 4 104607 Gastroenterology, Chinese PLA General Hospital, Beijing, China; 5 104607 Department of Gastroenterology, Chinese PLA General Hospital, Beijing, China; 6 104607 Gastroenterology, Chinese PLA General Hospital, Beijing, China

With the improvement and popularization of radiological and peroral cholangioscopy techniques, more and more polypoid lesions in the biliary duct system, including the common bile duct (CBD), common hepatic duct (CHD), and gallbladder, have been found 1 2 3 . Patients with polypoid lesions in the biliary duct system often faced a dilemma. Surgical treatment for those polypoid lesions was accompanied by relatively major trauma; on the other hand, follow-up observation comes with the risk of progression of the lesions. Therefore, our team developed a kind of snare with an electrocision function that can pass through the working tunnel of a peroral cholangioscope. In this study, we attempted the cholangioscopy-assisted endoscopic mucosal resection using the aforementioned snare through papillary support for a CBD lesion in a porcine model.

First, a single dumbbell-style papillary support 4 was placed in the lower CBD and papilla after biliary intubation ( Fig. 1 ). Second, the cholangioscope (eyeMax, 11 F; Micro-Tech, Nanjing, China) was inserted into the CBD ( Fig. 2 ). Third, a submucosal injection for a part of the CBD mucosa was performed using an injection needle under direct vision ( Fig. 3 ). Fourth, the specially designed snare was inserted into the CBD through the working tunnel of the cholangioscope, and electrocoagulation was performed ( Fig. 4 ). Fifth, a part of the CBD mucosa was resected successfully using the snare by the electrocision function ( Fig. 5 , Video 1 ). Finally, the papillary support was removed. No serious adverse event was encountered during the 1-week follow-up.

**Fig. 1:**
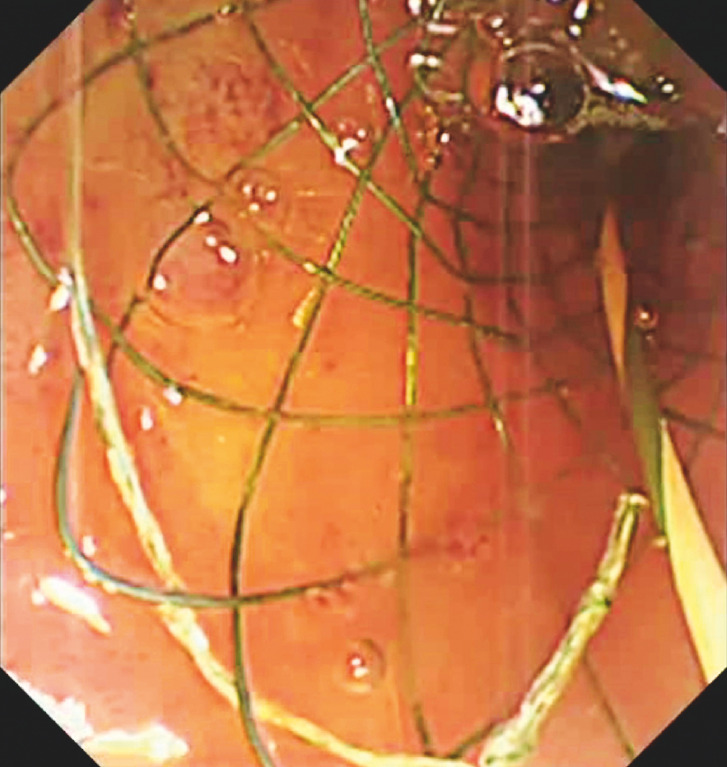
A single dumbbell-style papillary support was placed in the lower common bile duct (CBD) and papilla after biliary intubation.

**Fig. 2:**
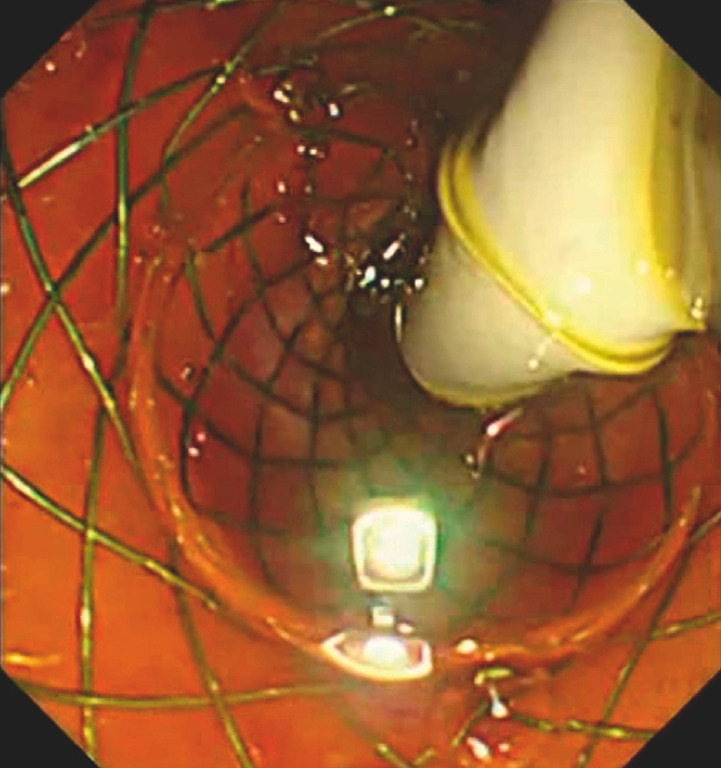
The cholangioscope was inserted into the CBD.

**Fig. 3:**
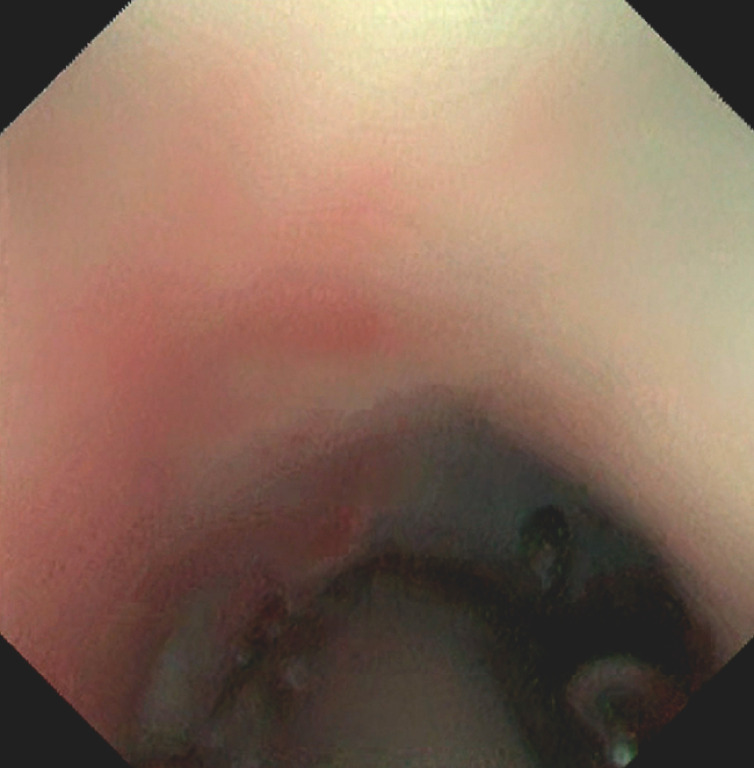
Submucosal injection for a part of the CBD mucosa was performed using an injection needle under direct vision.

**Fig. 4:**
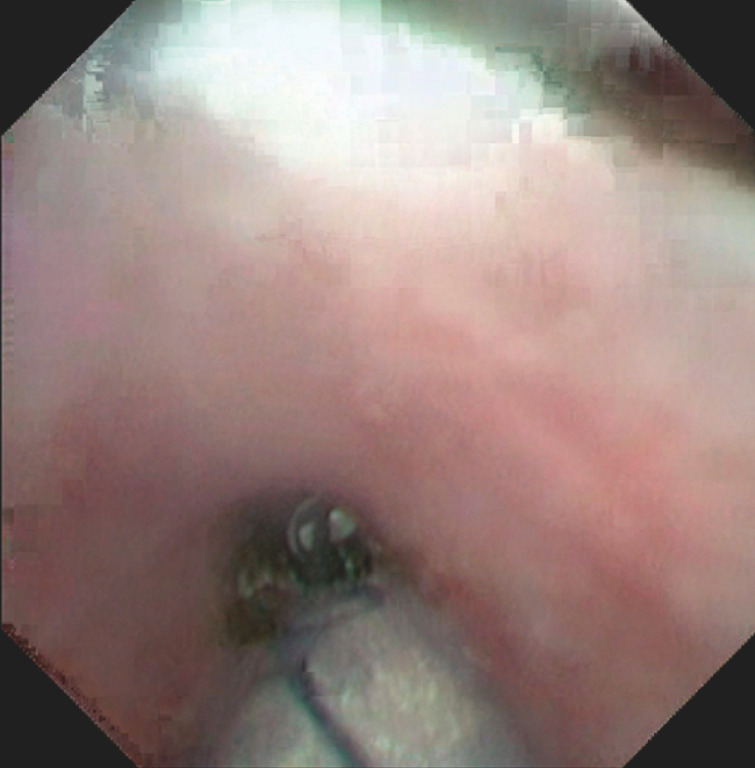
The specially designed snare was inserted into the CBD through the working tunnel of the cholangioscope, and electrocoagulation was performed.

**Fig. 5:**
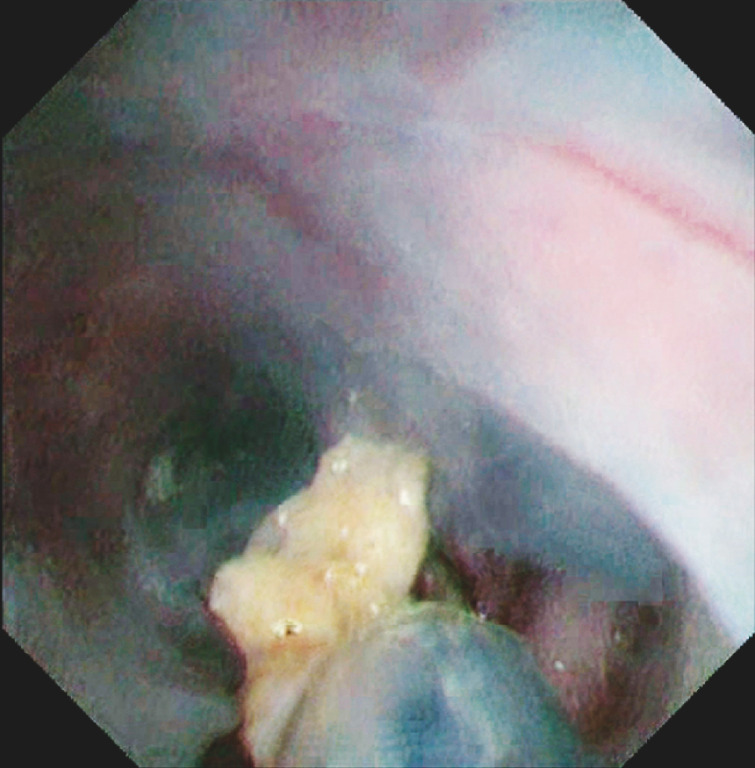
A part of the CBD mucosa was resected successfully using the snare by the electrocision function.

The procedures of cholangioscopy-assisted endoscopic mucosal resection for a common bile duct lesion through papillary support.Video 1

This study preliminarily confirmed the feasibility and safety of cholangioscopy-assisted endoscopic mucosal resection for lesions of the biliary duct system through papillary support in a porcine model, although further clinical studies are warranted.

Endoscopy_UCTN_Code_TTT_1AR_2AD

## Notes

Endoscopy E-Videos https://eref.thieme.de/e-videos
*E-Videos* is an open access online section of the journal *Endoscopy* , reporting on interesting cases and new techniques in gastroenterological endoscopy. All papers include a high-quality video and are published with a Creative Commons CC-BY license. Endoscopy E-Videos qualify for HINARI discounts and waivers and eligibility is automatically checked during the submission process. We grant 100% waivers to articles whose corresponding authors are based in Group A countries and 50% waivers to those who are based in Group B countries as classified by Research4Life (see: https://www.research4life.org/access/eligibility/ ). This section has its own submission website at https://mc.manuscriptcentral.com/e-videos .
